# The autoinhibitory CARD2-Hel2i Interface of RIG-I governs RNA selection

**DOI:** 10.1093/nar/gkv1299

**Published:** 2015-11-26

**Authors:** Anand Ramanathan, Swapnil C. Devarkar, Fuguo Jiang, Matthew T. Miller, Abdul G. Khan, Joseph Marcotrigiano, Smita S. Patel

**Affiliations:** 1 Robert Wood Johnson Medical School, Department of Biochemistry and Molecular Biology, Rutgers University, Piscataway, NJ 08854, USA; 2 Center for Advanced Biotechnology and Medicine, Department of Chemistry and Chemical Biology, Rutgers University, Piscataway, NJ 08854, USA

## Abstract

RIG-I (Retinoic Acid Inducible Gene-I) is a cytosolic innate immune receptor that detects atypical features in viral RNAs as foreign to initiate a Type I interferon signaling response. RIG-I is present in an autoinhibited state in the cytoplasm and activated by blunt-ended double-stranded (ds)RNAs carrying a 5′ triphosphate (ppp) moiety. These features found in many pathogenic RNAs are absent in cellular RNAs due to post-transcriptional modifications of RNA ends. Although RIG-I is structurally well characterized, the mechanistic basis for RIG-I's remarkable ability to discriminate between cellular and pathogenic RNAs is not completely understood. We show that RIG-I's selectivity for blunt-ended 5′-ppp dsRNAs is ≈3000 times higher than non-blunt ended dsRNAs commonly found in cellular RNAs. Discrimination occurs at multiple stages and signaling RNAs have high affinity and ATPase turnover rate and thus a high *k*_atpase_/*K*_d_. We show that RIG-I uses its autoinhibitory CARD2-Hel2i (second CARD-helicase insertion domain) interface as a barrier to select against non-blunt ended dsRNAs. Accordingly, deletion of CARDs or point mutations in the CARD2-Hel2i interface decreases the selectivity from ≈3000 to 150 and 750, respectively. We propose that the CARD2-Hel2i interface is a ‘gate’ that prevents cellular RNAs from generating productive complexes that can signal.

## INTRODUCTION

RIG-I like receptors, found in most cell types, are cytosolic sensors of viral RNAs. These receptors include RIG-I, MDA5 and LGP2, which are superfamily 2 RNA helicases/ATPases with the ability to recognize specific RNA features and trigger an immune response against a wide variety of commonly found RNA viruses (1–5). RIG-I recognizes 5′-triphosphate (5′ppp) and 5′pp (6–8) on base-paired and blunt-ended double-stranded (ds)RNAs (9,10) found in many viral genomes and their replication intermediates (11–16). These features are not present in cellular RNAs because of post-transcriptional modifications and the lack of an RNA-dependent RNA polymerase in mammalian cells. For example, mRNAs contain 5′ppp, but these are capped. Similarly, RNA stems are present in large folded RNAs and tRNAs, but these are not blunt-ended. The microRNAs are short double-stranded RNAs, but they contain 3′-overhangs formed during post transcriptional processing. Thus, RIG-I is constantly exposed to non-blunt ended RNAs in the cytoplasm, but the receptor remains silent, a crucial property for preventing inappropriate activation of the immune system (17,18). Biochemical studies have measured RNA affinities and ATPase turnover rates of blunt-ended 5′ppp and 5′OH dsRNAs (19–21), but a systematic study of the effect of RNA-end modifications on RNA affinity, ATPase activity and signaling is lacking. Such studies, for example, of the immune receptor PKR (Protein kinase R) yielded insights into cellular modifications that prevent self RNAs from activating PKR (22,23).

**Figure 1.**
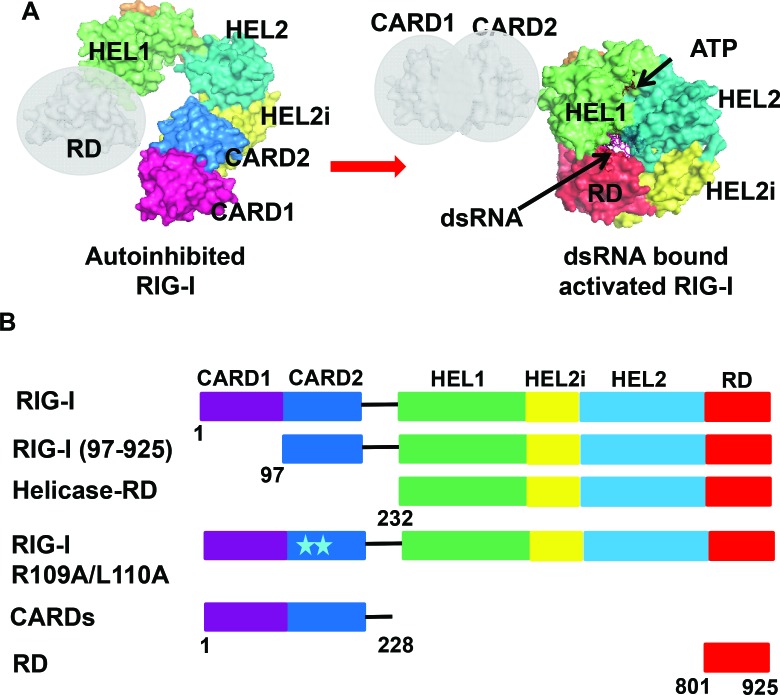
Structural model of RIG-I activation and schematics of RIG-I constructs used in this study. **(A)** Autoinhibited RNA free RIG-I modeled using structures of full length duck RIG-I (PDB ID: 4a2w) and duck RIG-I RD (PDB ID: 4a2v). Active dsRNA bound RIG-I modeled using structures of helicase-RD (PDB ID: 3tmi) and duck RIG-I CARD domains (PDB ID: 4a2w). **(B)** Schematic of human-RIG-I full length domains and the different deletion and mutant constructs used in this study. Models were generated using Pymol (Version 1.7.4., Schrödinger, LLC).

The current model of RIG-I activation is based on three key crystal structures (19,24,25). The RIG-I structure without RNA and ATP shows that the Hel1 and Hel2 helicase subdomains are in an open conformation and the N-terminal tandem caspase activation and recruitment domains (CARDs) are sequestered by the Hel2i (helicase insertion) subdomain (24). The open helicase conformation explains the lack of ATP hydrolysis activity and the sequestered CARDs explains why RIG-I without RNA is autoinhibited for downstream signaling. The structure of RIG-I without CARDs but with dsRNA and ATP analogue shows that the Hel1 and Hel2 are closed around ATP and dsRNA and the C-terminal RD is interacting with the 5′-end of the dsRNA (19). Comparison of the RIG-I structures with and without RNA suggests that RIG-I activation involves breaking of the CARD2-Hel2i interactions (Figure 1A). However, the role of this interface in RNA selection by RIG-I is poorly understood.

In this study, we use fluorescence and ATPase-based RNA binding assays to quantify the RNA equilibrium dissociation constant (*K*_d_) and the ATPase turnover rate (*k*_atpase_) of RIG-I complexes with blunt-ended and non-blunt ended dsRNAs. We correlate these parameters to the ability of the RNA molecule to stimulate cell-based signaling activity and find a good correlation between signaling and *k*_atpase_/*K*_d_ ratio and *K*_d_, but not with the *k*_atpase_. Non-blunt-ended dsRNAs without 5′ppp and with a 5′-overhang have the lowest RNA affinity and ATPase turnover rate and signal poorly whereas 3′-overhang is not as deleterious. By carrying out studies with full-length RIG-I (referred to as RIG-I from here on), RIG-I domains and interface mutant, we show that CARD2-Hel2i interface plays a key role in RNA selection by acting as a ‘gate’ to allosterically regulate both RNA affinity and ATPase activity. Thus, the molecular switch used in autoinhibition also governs RNA selection.

## MATERIALS AND METHODS

### RNA substrates

HPLC purified synthetic ssRNAs containing 5′OH from Dharmacon (ThermoFisher) were deprotected and resuspended in 20 mM potassium phosphate buffer pH 7.0. The RNA concentration was determined in 7M guanidine HCl using the NanoDrop spectrophotometer at A_260_. All 5′ppp containing ssRNAs were obtained from Biosynthesis. Duplex RNAs were prepared by mixing complementary ssRNAs in a 1:1.1 ratio, heating to 95°C for 1 min and slow cooling to 4°C. Fluorescent RNAs were synthesized as ssRNA with fluorescein attached to 5′ or 3′ terminus. All RNAs were checked by sequencing gel electrophoresis in 7 M urea for purity before use.

### Protein expression and purification

All the RIG-I constructs were sub-cloned into a modified pET28b vector with N-terminal SUMO fusion. Human RIG-I (1–925), Helicase-RD (232–925), and 2^nd^ CARD-Helicase-RD (97–925), CARDs (1–228) were overexpressed in *E. coli* strain Rosetta (DE3) (Novagen) as soluble proteins. The isolation of pure proteins involved three chromatographic steps: affinity column (Ni^2+^-nitrilotriacetate, Qiagen), hydroxyapatite column (CHT-II, Bio-Rad) and heparin sepharose column (GE Healthcare). An additional gel-filtration chromatography step (Hiload 16/26 Superdex 200, GE Healthcare) was added to purify RIG-I. Purified helicase-RD was further dialyzed overnight at 4°C into 50 mM HEPES pH 7.5, 50 mM NaCl, 5mM DTT, 10% glycerol, snap frozen in liquid nitrogen and stored at –80°C as reported previously (19). The RD (801–925) was expressed in *E. coli* BL21 Star (DE3) cells and the soluble fraction was purified to homogeneity using a Ni^2+^affinity column, cation exchange (HiTrap SP, GE Healthcare) and gel filtration chromatography.

### Preparation of the CARD2 double substitutions of RIG-I R109A/L110A

The double mutation in RIG-I gene was introduced using the QuikChange II XL site-directed mutagenesis kit from Agilent Technologies. The mutagenic primers used were:

5′-GGAGTATAGATTACTTTTAAAAGCTGCACAACCAGAATTTAAAACC-3′ (Forward)

5′-GGTTTTAAATTCTGGTTGTGCAGCTTTTAAAAGTAATCTATACTCC-3′ (Reverse)

Purification of the mutant was carried out using the same protocol as RIG-I.

### ATP hydrolysis

The ATP hydrolysis assays were performed in 1X Buffer-A at 15°C unless otherwise mentioned. 1X Buffer A: 50 mM MOPS-Na (pH 7.4), 5 mM MgCl_2_, 5 mM DTT, 0.01% Tween 20 (19). The ATP hydrolysis time course (0–60 min) was measured using 5 nM protein for blunt-ended dsRNA and 25 nM protein for non-blunt ended dsRNA, 1 mM ATP spiked with [*γ*-^32^P]ATP and RNA substrates (1 μM) in Buffer A at 15°C or 37°C. The reactions were stopped at desired time points using 4N HCOOH and analyzed by PEI-Cellulose-F TLC (Merck) developed in 0.4 M potassium phosphate buffer (pH 3.4). The TLC plates were exposed to a phosphorimager plate, which was imaged on a Typhoon phosphor-imager and quantified using ImageQuant software. The ATPase turnover rate was determined from the plots of [Pi] produced versus time and dividing the ATPase turnover rate by the respective enzyme concentration.

### Fluorescence anisotropy titrations

Fluorescence anisotropy measurements were carried out using FluoroMax-4 spectrofluorimeter (Horiba JobinYvon) in Buffer A as previously described (26). Fluorescein labeled blunt-ended dsRNAs (1 or 2 nM) or non-blunt ended dsRNAs (40 nM) were titrated with increasing protein and fluorescein anisotropy was measured at 15°C with excitation at 494 nm and emission at 516 nm. The observed fluorescence anisotropy (*r*_obs_) was plotted as a function of protein concentration (P_t_) and fit to Equations (1) and (2) to obtain the equilibrium dissociation constant, *K*_d_.

(1) \begin{equation*} r_{obs} = r_b f_b + r_f (1 - f_b ) \end{equation*}

Where, *r*_f_ and *r*_b_ are the anisotropy values of free RNA and of the complex, *f*_b_ is the fraction of RNA bound in the protein-RNA complex and *f*_b_ = [PR]/[R_t_] (PR is the concentration of the protein-RNA complex and R_t_ is the total RNA concentration).

(2) \begin{equation*} {[{\rm PR}] =} {([{\rm P}_{\rm t} ] + [{\rm R}_{\rm t} ] + K_{\rm d} ) - \frac{{\sqrt {([{\rm P}_t ] + [{\rm R}_{\rm t} ] + K_{\rm d} )^2 - 4[{\rm P}_{\rm t} ][{\rm R}_{\rm t} ]} }}{2}} \end{equation*}

The reported RNA *K*_d_ values were consistently observed in titrations repeated 2 to 3 times.

### Determination of *K*_d. app_ under ATPase cycling conditions

The ATPase turnover rate was measured at constant protein (5 nM or 25 nM) and increasing RNA (0.1 nM–2 μM) in the presence of 1 mM ATP (spiked with [*γ*-^32^P] ATP). A time course (0–60 min) of the ATPase reactions was performed in Buffer A at 15°C or 37°C and the initial velocities were plotted as a function of time to obtain the ATPase rate. The error bars were obtained from the fit of the time-course of the ATPase reactions. The quenched reactions were analyzed as indicated above to obtain the ATPase turnover rates that were plotted as a function of RNA concentration and fitted to hyperbolic equation (3) or quadratic equation (2) where observed ATPase = maximum ATPase x [PR]/[Pt], where [Pt] is total protein concentration.

(3) \begin{equation*} [{\rm PR}] = \frac{{[{\rm S}]}}{{K_{{\rm d,app}} + [{\rm S}]}} \end{equation*}

### IFN-β reporter signaling assays

HEK293T cells were grown in 6-well plates to 60% confluence and co-transfected with firefly luciferase reporter plasmid (2.5 μg), Renilla luciferase reporter plasmid (500 ng) and a plasmid carrying the wild type (wt) RIG-I gene under CMV promoter or an empty plasmid (2 μg). The firefly luciferase gene is under the interferon β promoter and the Renilla luciferase plasmid is under the constitutively active TK promoter. The plasmid transfections were carried out with X-tremeGENE HP DNA Transfection Reagent (Roche). Cells were re-plated in 96-well plates the next day at 2 × 10^4^ cells/well density and transfected with each of the RNA ligands (900 nM final concentration/well) or Poly I:C (700 ng/well) using Lipofectamine transfection reagent (Life Technologies). After 20 h the activities of firefly and Renilla luciferases were measured sequentially with the Dual-Luciferase reporter assay kit (Promega). Data were collected in quadruplicate sets and the relative luciferase activities were calculated. The error bars represent the standard error of the mean (SEM).

## RESULTS

### RNA ligands

To circumvent the heterogeneity associated with *in vitro* transcribed RNAs with questionable RNA-ends (9,10), we used chemically synthesized 10-nt RNAs with defined RNA-end modifications. These included blunt-ended dsRNAs with 5′OH or 5′ppp, 3′-end 2-nt (nucleotide) ssRNA overhangs with 5′-ppp or 5′-OH (3′-ovg), and 5′-end 2-nt ssRNA overhangs with 5′ppp or 5′OH (5′-ovg) (Supplementary Table S1). We used short dsRNAs to avoid complications from two RIG-I molecules binding to each end of the dsRNA, thus assuring measurement of *K*_d_ values and ATPase rates of the 1:1 RIG-I/RNA complexes. The RNA binding and ATPase studies were carried out at 15°C to avoid complications from RNA duplex integrity. However, we also studied longer dsRNAs and hairpin RNAs. Using this panel of dsRNAs of the same sequence, but different end-modifications, we studied RNA binding and ATPase activation of RIG-I, Helicase-RD lacking the CARDs, RIG-I lacking just the first CARD (residues 97–925), the C-terminal repressor domain (RD) and the CARD2-Hel2i interface mutant (Figure 1B). These studies enabled us to understand the contributions of each of the RIG-I domains and end-modifications to RNA selection.

### Interactions of RIG-I with blunt-ended dsRNAs

**Figure 2.**
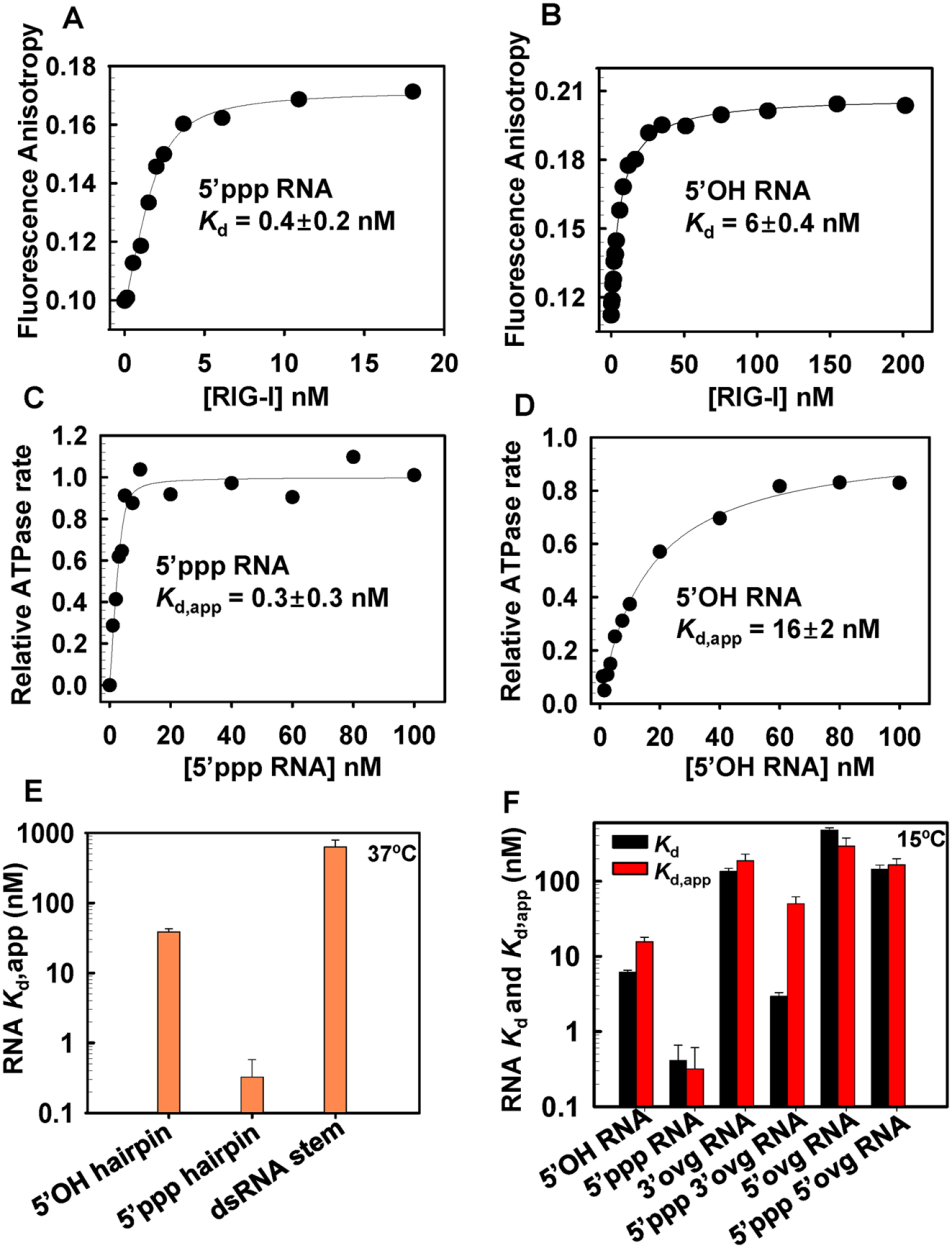
*K_d_* values of full-length RIG-I complexes with blunt-end and non-blunt ended dsRNA in the absence and presence of ATP hydrolysis. **(A–B)** Fluorescence anisotropy of 5′ fluorescein labeled dsRNA with 5′ppp or 5′OH (2 nM) was measured after addition of increasing amounts of RIG-I. The dissociation constant (*K*_d_) was determined from fitting the data to Equations (1) and (2) (Experimental methods). **(C–D)** The ATPase turnover rates of RIG-I (5 nM) was measured with increasing concentration of 5′ppp or 5′OH RNA. The data were fit to the quadratic equation to obtain the apparent dissociation constants (*K*_d_,_app_). **(E)** The *K*_d_,_app_ of RIG-I complexes with the indicated hairpin RNAs were obtained from the ATPase based titrations at 37°C in Buffer A. **(F)** The *K*_d_ from the anisotropy assay (black bars) and *K*_d_,_app_ from the ATPase assay at 15°C (red bars) for RIG-I complexes with the indicated RNAs. Errors are standard errors from the fittings.

The first step in RNA recognition and selection is RIG-I binding to the RNA. This step is characterized by the equilibrium dissociation constant (*K*_d_), which was estimated by fluorescence anisotropy/intensity based titrations that are non-disruptive, providing accurate *K*_d_ values, especially of weak complexes. The presence of fluorescein does not perturb binding to RIG-I (Supplementary Figure S1). The 5′-end fluorescein labeled dsRNA (with 5′ppp or 5′OH at the other end) was titrated with increasing concentration of RIG-I (Figure 2A, B) providing binding curves that fit well to the 1:1 binding model (Equations (1) and (2)). The *K*_d_ values from these experiments showed that RIG-I has a high affinity for the blunt-ended 5′ppp RNA (*K*_d_ = 0.4 ± 0.2 nM). Replacing 5′ppp with 5′OH results in a ≈15-fold lower affinity (*K*_d_ = 6 ± 0.4 nM) (Table 1).

**Table 1.** ATPase rates, RNA *K*_d,app_ and RNA *K*_d_ of RIG-I, Helicase-RD and RD with 10-bp dsRNAs (15°C)

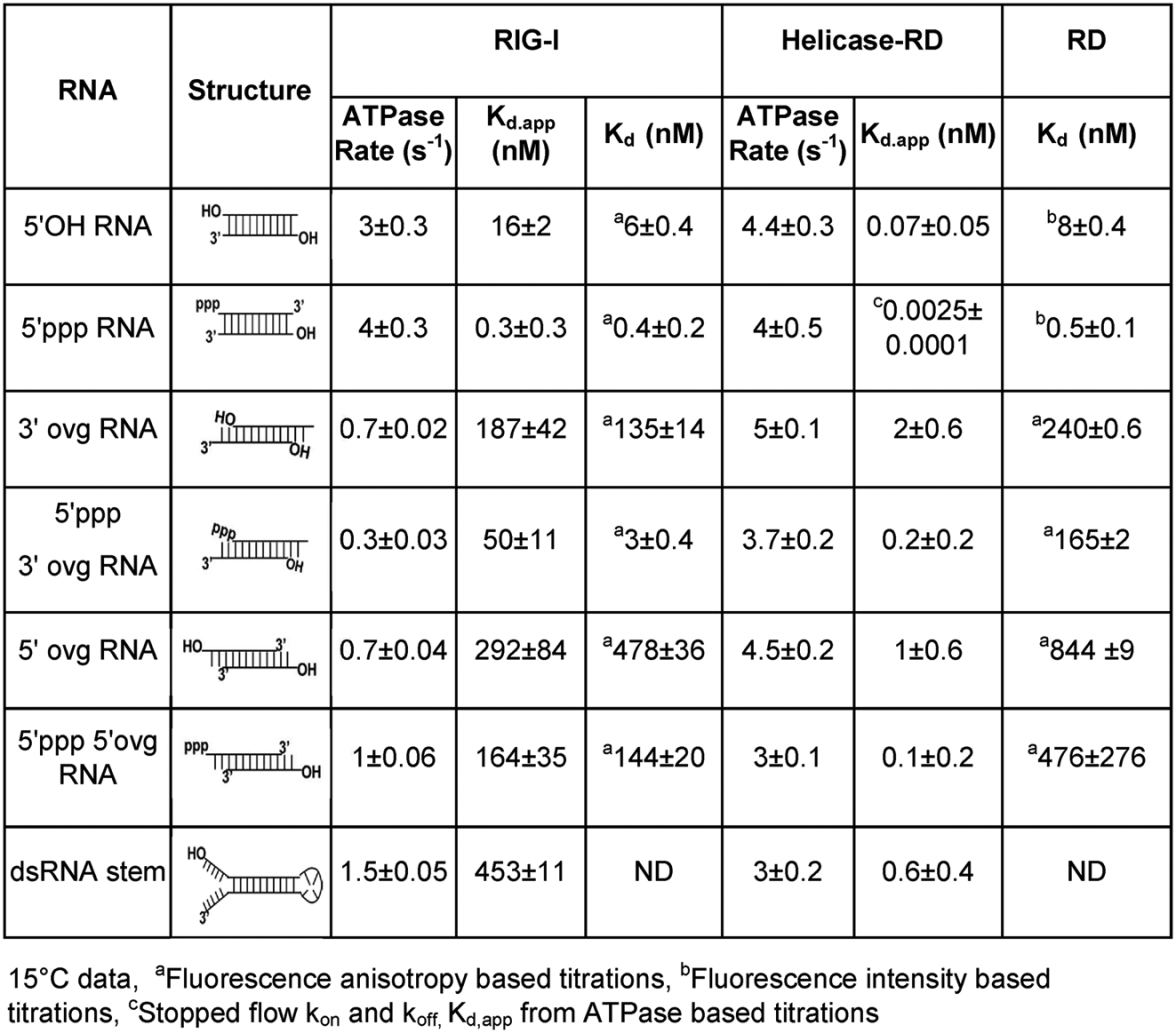							

*In vivo*, RNA binding occurs in the presence of ATP; hence, we measured the RNA binding under ATPase cycling conditions by measuring the ATPase turnover rate as a function of increasing RNA concentration (*K*_d,app_). The ATPase turnover rate versus RNA concentration curves fit well to the 1:1 (RIG-I:RNA) binding stoichiometry (Figure 2C, D). RNA affinity measured by the ATPase-based titrations report functional binding events that result in ATP hydrolysis, whereas fluorescence anisotropy measures all physical association events, including those that do not result in ATP hydrolysis. Thus, we do not expect the two methods to provide the same *K*_d_ values. However, the RNA *K*_d,app_ of the 5′ppp RNA under the ATPase cycling conditions was the same as the *K*_d_ in the absence of ATP, but the *K*_d,app_ of the 5′OH RNA was ≈2.5 times higher under the ATPase cycling conditions relative to conditions without ATP (Figure 2B, D and Table 1). Similar results were obtained with the hairpin 5′ppp and 5′OH RNAs (Figure 2E, Supplementary Table S2). Thus, our results suggest that RIG-I's selectivity for 5′ppp RNA versus 5′OH RNA is slightly better under ATPase cycling conditions.

### RIG-I binds to non-blunted dsRNAs

We next determined whether RIG-I binds to non-blunt ended dsRNAs. The fluorescence anisotropy titrations showed that all the non-blunt ended dsRNAs bind to RIG-I but with varying *K*_d_ values that ranged from as low as 3 nM to 478 nM (Figure 2F, Table 1). The 5′ppp 3′ovg RNA forms the tightest complex (3 nM), and the 5′OH 5′ovg dsRNA and hairpin RNA with two overhangs formed weak complexes (≈450 nM). Under ATPase cycling conditions, the *K*_d,app_ values of all non-blunt ended dsRNAs are similar to the K­_d_ values in the absence of ATP, except for the 5′ppp 3′ovg RNA (Figure 2F, Table 1). The binding of 5′ppp 3′ovg RNA under ATPase cycling conditions is about 16-fold weaker than without ATP, which indicates that ATP hydrolysis modulates the binding affinity of the 5′ppp 3′ovg RNA, as we observe above with the 5′OH RNA. However, why ATPase activity affects the binding affinity of only these two RNAs is not understood. RIG-I has no detectable binding affinity for short ssRNAs with or without 5′ppp (Supplementary Figure S2A, S2B). Overall, our results indicate that non-blunt-ended dsRNAs bind to RIG-I, but the RNA end modification influences the RNA binding affinity.

### The C-terminal domain (RD) discriminates between RNA ligands

**Figure 3.**
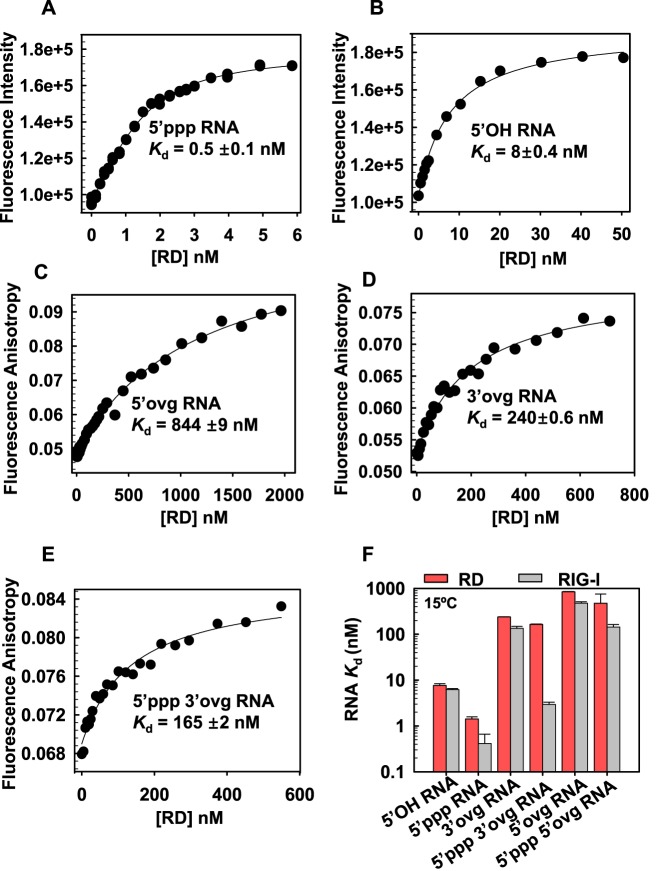
*K*_d_ values of the C-terminal RIG-I RD complexes with blunt-end and non-blunt ended dsRNAs. **(A–B)** Fluorescence intensity of 3′ fluorescein labeled dsRNA with 5′ppp or 5′OH (2 nM) was measured after addition of increasing concentration of the RD protein. The data were fit to Equation (3) to obtain the dissociation constant (*K*_d_) values. **(C–E)** Fluorescence anisotropy of 5′ fluorescein labeled overhang dsRNAs (40 nM) was measured with increasing concentrations of RD and data were fit to Equations (1) and (2) to obtain the *K*_d_ values. **(F)** The bar chart compares the *K*_d_ values of RD and RIG-I complexes with the indicated RNAs. Standard errors from fitting are shown.

The C-terminal RD domain of RIG-I is considered a sensor of RNA ends (24,27,28). To determine the contribution of the RD to RNA selectivity, we used fluorescence intensity/anisotropy based titrations to quantify the *K*_d_ values of the RD-RNA complexes. The binding of blunt-ended dsRNAs to RD was reliably measured using fluorescence intensity changes with fluorescein at the 3′-end. The RD forms tight complexes with blunt-ended 5′ppp RNA (Figure 3A) and 5′OH RNA (Figure 3B) with *K*_d_ values almost identical to those of the full-length RIG-I (Table 1).

The RD also binds to non-blunt ended dsRNAs; the 3′ovg RNAs bind more tightly as compared to the 5′ovg RNAs, and those with 5′ppp bind more tightly than dsRNAs with 5′OH (Figure 3 C-F, Table 1). This is similar to the full-length RIG-I; the exception is 5′ppp 3′ovg RNA, which binds to the RD with 165 ± 2 nM *K*_d_ (Figure 3F) as opposed to 3 ± 0.4 nM *K*_d_ to the full-length RIG-I. This indicates that both RD and the helicase domain of RIG-I are required for the observed high affinity binding of the 5′ppp 3′ovg RNA.

### Removal of CARDs increases the binding affinity for all RNAs

**Figure 4.**
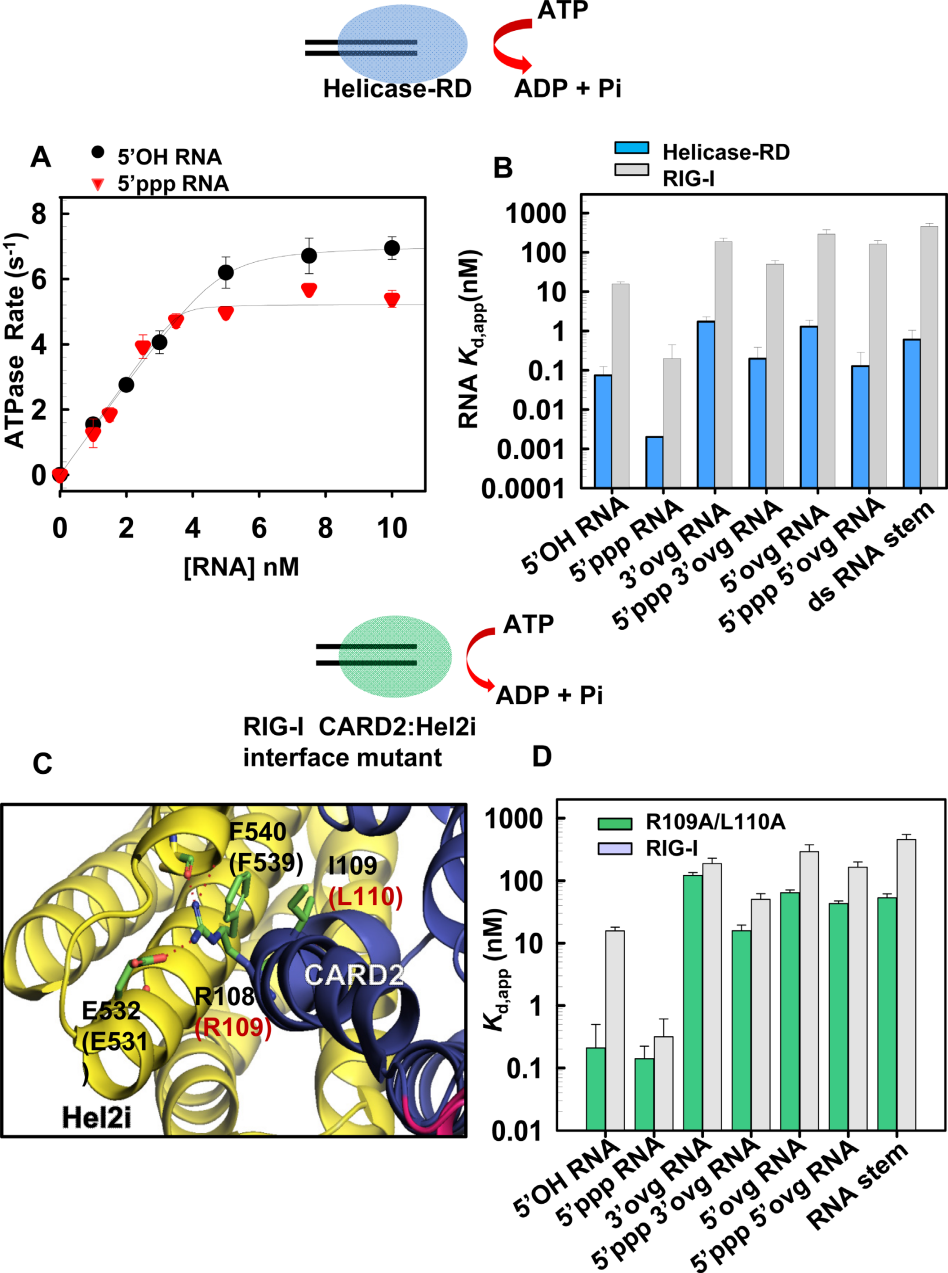
Loss of RNA binding selectivity upon removal of CARDs or mutation in the CARD2-Hel2i interface. **(A)** Helicase-RD (5 nM) was titrated with increasing concentrations of 5′OH RNA (black circles) or 5′ppp RNA (red inverted triangles) and the ATPase turnover rates were measured at 15°C in Buffer A. The binding curves show stoichiometric 1:1 binding of Helicase-RD and RNA. **(B)** Bar Chart compares the apparent dissociation constant *K*_d,app_ of Helicase-RD (blue bars) for the various RNAs are shown in comparison to RIG-I (gray bars). The *K*_d_ of Helicase-RD for 5′ppp RNA was determined from the *off* and *on* rates. Standard errors from the fitting are shown. **(C)** The CARD2 (blue) and Hel2i (yellow) interface residues in duck RIG-I and the corresponding residues inhuman RIG-I (in parentheses) are shown. CARD2 residues R109 and L110 interact with Hel2i residues E531 and F539, respectively. **(D)** Bar Chart compares the *K*_d_,_app_ values of R109A/L110A RIG-I (green bars) complexes with indicated RNAs are shown in comparison to RIG-I (gray bars). Standard errors from fitting are shown (also see Supplementary Table S3).

The CARDs are responsible for interacting with the downstream adapter proteins to relay the signal, however their role in RNA discrimination is not known. Deleting the CARDs (Helicase-RD) increases the binding affinity for both 5′ppp and 5′OH blunt-ended dsRNAs. Data fitting to the quadratic equation provided *K*_d,app_ of 0.07 nM for the 5′OH RNA (Figure 4A), but the affinity for 5′ppp RNA was too tight to estimate by this method. Therefore, kinetic *off-rate* and *on-rate* were used to determine the *K*_d_ value of the 5′ppp RNA complex. The ratio of the *off-rate* (1.5 × 10^−3^ s^−1^) to the *on-rate* (6 × 10^8^ M^−1^s^−1^) yielded a *K*_d_ of 2.5 pM for 5′ppp RNA (Supplementary Figure S3). Thus, deletion of CARDs increases the affinity of RIG-I for blunt-ended dsRNAs by 150–250-fold, indicating that the CARDs regulate RNA affinity. We found that adding CARDs *in trans* to the Helicase-RD weakened RNA affinity by about 2-fold (Supplementary Figure S4A).

Surprisingly, the Helicase-RD construct of RIG-I binds to all non-blunt ended dsRNAs with a high affinity in the sub-nanomolar range (Figure 4B). The *K*_d,app_ values of non-blunt ended complexes range from ≈0.1 nM for 5′ppp and 3′- and 5′-ovg RNA complexes and ≈1.5 nM for 5′OH and 3′- or 5′-ovg RNA (Table 1). The RNA hairpin with two overhangs mimicking an RNA stem also binds to the Helicase-RD with a high affinity (*K*_d,app_ of 0.6 ± 0.4 nM). Similarly, ssRNAs binds to the Helicase-RD with *K*_d,app_ of ≈1600 ± 500 nM for 5′OH ssRNA and ≈500 ± 100 nM for the 5′ppp ssRNA but does not bind to RIG-I (Supplementary Figure S2A, S2B). These results indicate that the CARDs are preventing RIG-I from interacting with the non-blunt ended dsRNAs and ssRNAs. Interestingly, the ratio of the *K*_d_ values of RIG-I and Helicase-RD shows that the presence of CARDs is critical for discriminating against binding of dsRNAs with 5′ovg and stem RNAs (Supplementary Figure S5A).

### The CARD2-Hel2i interface acts as a selectivity gate

The duck RIG-I structure shows that CARDs are interacting with Hel2i when RIG-I is not bound to RNA and ATP (24). To investigate whether CARD2-Hel2i interface is responsible for weakening RNA binding, we designed several CARD2-Hel2i interface breaking mutants using the structure of duck RIG-I (24) and amino acid homology between duck and human RIG-I CARD2. We mutated residues in CARD2 rather than Hel2i to avoid pleiotropic effects arising from mutating the helicase domain that interacts with RNA and ATP (Figure 4C). After several trials with different mutants, we were able to produce soluble double alanine substitutions of R109 and L110 (R109A/L110A).

We measured the *K*_d,app_ of complexes of CARD2-Hel2i interface mutant R109A/L110A with blunt-ended and non-blunt ended dsRNA using the ATPase based assays (Figure 4D). The R109A/L110A mutant binds both 5′ppp and 5′OH blunt-end dsRNAs with similar *K*_d_ values (≈0.2 nM), indicating that a disruption the CARD2-Hel2i interface results in a loss of discrimination against 5′OH RNA. The CARD2 interface mutant also binds to non-blunt-ended dsRNAs with 1.5- to 4.6-fold higher affinities relative to wild type RIG-I (Table 2). Therefore, mutating the CARD2-Hel2i interface increases the affinity of RIG-I for the non-blunt ended dsRNAs, similar to the effect seen after deletion of the CARDs.

**Table 2.** ATPase Rates and *K*_d_._app_ of RIG-I R109Al110A for the different RNAs

RNA	R109AL110A RIG-I	
	ATPase Rate (s^–1^) 15ºC	*K*_d,app_(nM) 15ºC
5′OH RNA	2 ± 0.1	0.2 ± 0.3
5′ppp RNA	2 ± 0.1	0.1 ± 0.1
3′ ovg RNA	1.6 ± 0.1	121 ± 14
5′ppp 3′ ovg RNA	0.9 ± 0.1	16 ± 4
5′ ovg RNA	1 ± 0.03	64 ± 7
5′ppp 5′ovg RNA	1 ± 0.06	43 ± 5
dsRNA stem	1.2 ± 0.1	53 ± 9

### Regulation of the ATPase activity of RIG-I by end-modification in dsRNA

**Figure 5.**
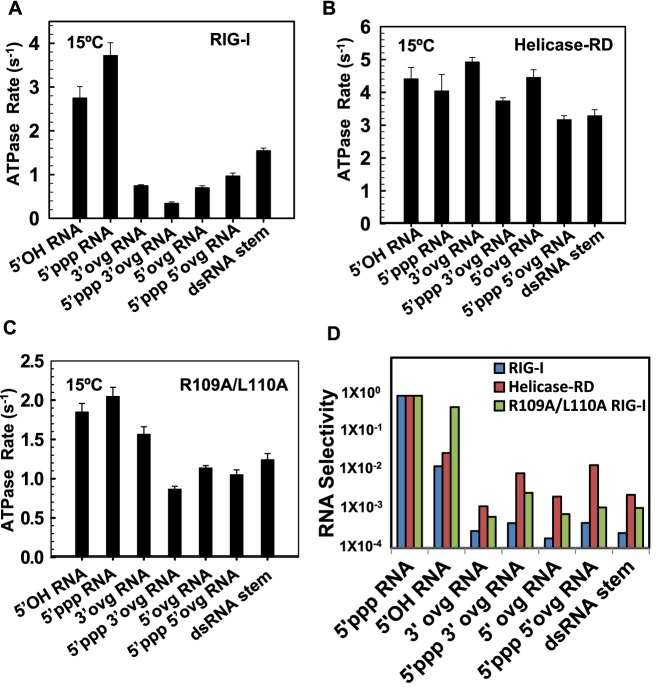
The ATPase activity and RNA selectivity of full-length RIG-I, Helicase-RD and R109A/L110A RIG-I mutant. **(A–C)** The bar chart compares the indicated RNA stimulated ATPase turnover rates of RIG-I (A) and Helicase-RD (B) and R109A/L110A (C) at 1 mM ATP and 1 μM of RNAs measured at 15°C in Buffer A. Errors from two independent experiments are shown. **(D)** The bar chart compares the RNA selectivity of RIG-I, Helicase-RD and R109A/L110A for the indicated RNAs normalized to the selectivity for 5′ppp RNA (Also see Supplementary Table S5).

Although the exact role of RIG-I's ATPase activity is not known, its importance in mediating RIG-I's signaling function is well documented. Mutations that abrogate or compromise RIG-I's ATPase function lead to defects in their signaling activity (29–32). We find that at 1 mM ATP, the RIG-I's ATPase turnover rate with 5′ppp RNA is the highest, followed by 5′OH RNA, and the non-blunt-ended dsRNAs have 4–10-fold lower ATPase turnover rate as compared to the 5′ppp RNA (Figure 5A, Table 1). The same trend is observed also at saturating ATP concentration; the ATPase *k*_cat_ values of non-blunt ended RNAs on an average are ≈20-fold lower than the ATPase *k*_cat_ of 5′ppp RNA and the ATPase *k*_cat_/*K*_m_ values are 5–20-fold lower (Supplementary Figure S5B and Supplementary Table S3). Interestingly, the ATP *K*_m_ values do not show a clear trend and remain essentially constant (Supplementary Table S3). The lower ATPase turnover rates of RIG-I with non-blunt ended RNAs is not due to weak RNA binding, because these reactions were carried out at 1 μM dsRNA concentration, which is well above the *K*_d_ values of these complexes.

Deletion of CARDs or specific mutations of the CARD2-Hel2i interface has a significant effect on the relative ATPase turnover rates of RIG-I with blunt-ended and non-blunt ended dsRNAs. Interestingly, the ATPase turnover rate, ATPase *k*_cat_, and the *k*_cat_/*K*_m_ values of the Helicase-RD are almost the same with blunt-ended and non-blunt-ended dsRNAs, differing only by about 2-fold (Figure 5B, Supplementary Figure S5C, Table S3 and Table 1). Similarly, the ATPase turnover rate of the R109A/L110A (CARD2-Hel2i interface mutant) is stimulated almost equally well by blunt-ended and non-blunt ended dsRNAs and the difference between the two types of RNAs is only about 2-fold (Figure 5C, and Table 2). These results indicate that CARDs and specifically the CARD2-Hel2i interface acts as a selectivity gate to regulate RNA affinities and prevent non-blunt ended dsRNAs from generating ATPase competent complexes.

### Selectivity of RIG-I for blunt-ended *versus* non-blunt-ended dsRNAs

To quantitate the selectivity of RIG-I for the 5′ppp RNA in a scenario where RIG-I is exposed to a pool of non-blunt-ended dsRNAs, we calculated the *k*_atpase_/*K*_d,app_ for all dsRNAs and then took the ratio of *k*_atpase_/*K*_d,app_ of the competing RNA ligand and the 5′ppp RNA. The *k*_atpase_/*K*_d,app_ is the initial slope of the ATPase rate versus RNA concentration titration curves and this parameter is analogous to the catalytic efficiency of a substrate providing a measure of how efficiently an RNA binds and activates the ATPase activity. The *k*_atpase_/*K*_d,app_ is the best parameter to compare the various RNAs, because the values of the individual parameters *k*_atpase_ and *K*_d,app_ are affected by non-productive complexes that do not hydrolyze ATP but their ratio *k*_atpase_/*K*_d,app_ is not (33). The *k*_atpase_/*K*_d,app_ of competitor RNA over that of the 5′ppp RNA is termed the RNA selectivity, and it shows that RIG-I chooses 5′ppp dsRNA on an average ≈3000 times over the non-blunt-ended dsRNAs (Figure 5D, Supplementary Table S4). When CARDs are removed, the RNA selectivity goes down to ≈150, and the CARD2-Hel2i interface mutant shows an intermediate RNA selectivity of ≈750.

### Signaling potential of dsRNAs with different end-modifications

Having measured the biochemical parameters of RIG-I complexes with dsRNAs of various end-modifications, we tested their signaling potential using IFN-β luciferase reporter assay in HEK293T cells. There was no endogenous RIG-I expression in the HEK293T cells, but expression of RIG-I from a transfected plasmid produced a detectable background signal in the absence of transfected RNA ligand, which was corrected (Supplementary Figure S6A). The 10-bp dsRNAs did not induce signaling possibly because of the instability of short dsRNAs at 37°C conditions of the cell-based assays; therefore, we prepared 23-bp dsRNAs of the same sequence and containing the same end-modifications as the short dsRNAs. To prevent a second RIG-I molecule from binding to the dsRNA at the other end, we introduced three base pairs of dsDNA at one end, since RIG-I is not capable of binding DNA. We used ATPase measurements at 37°C to measure the *K*_d,app_ and *k*_atpase_ of RIG-I with this entire set of 23-bp dsRNAs (Table 3). Interestingly, both the *K*_d,app_ and *k*_atpase_ of RIG-I shows the same trend with the short 10-bp and the longer 23-bp dsRNAs for different end-modifications. With both short and long dsRNAs, the 5′ppp RNA forms the tightest complex and the 5′ ovg RNA the weakest, and the 5′OH RNA binds with almost the same affinity as the 5′ppp 3′ovg RNA. Similarly, the blunt-ended RNAs stimulate the ATPase activity of RIG-I to a greater extent than the non-blunt ended dsRNAs.

**Table 3.** ATPase rates, *K*_d,app,_*k*_atpase_/*K*_d.app_ and Signaling Activity of RIG-I with 23-bp dsRNAs (37°C).

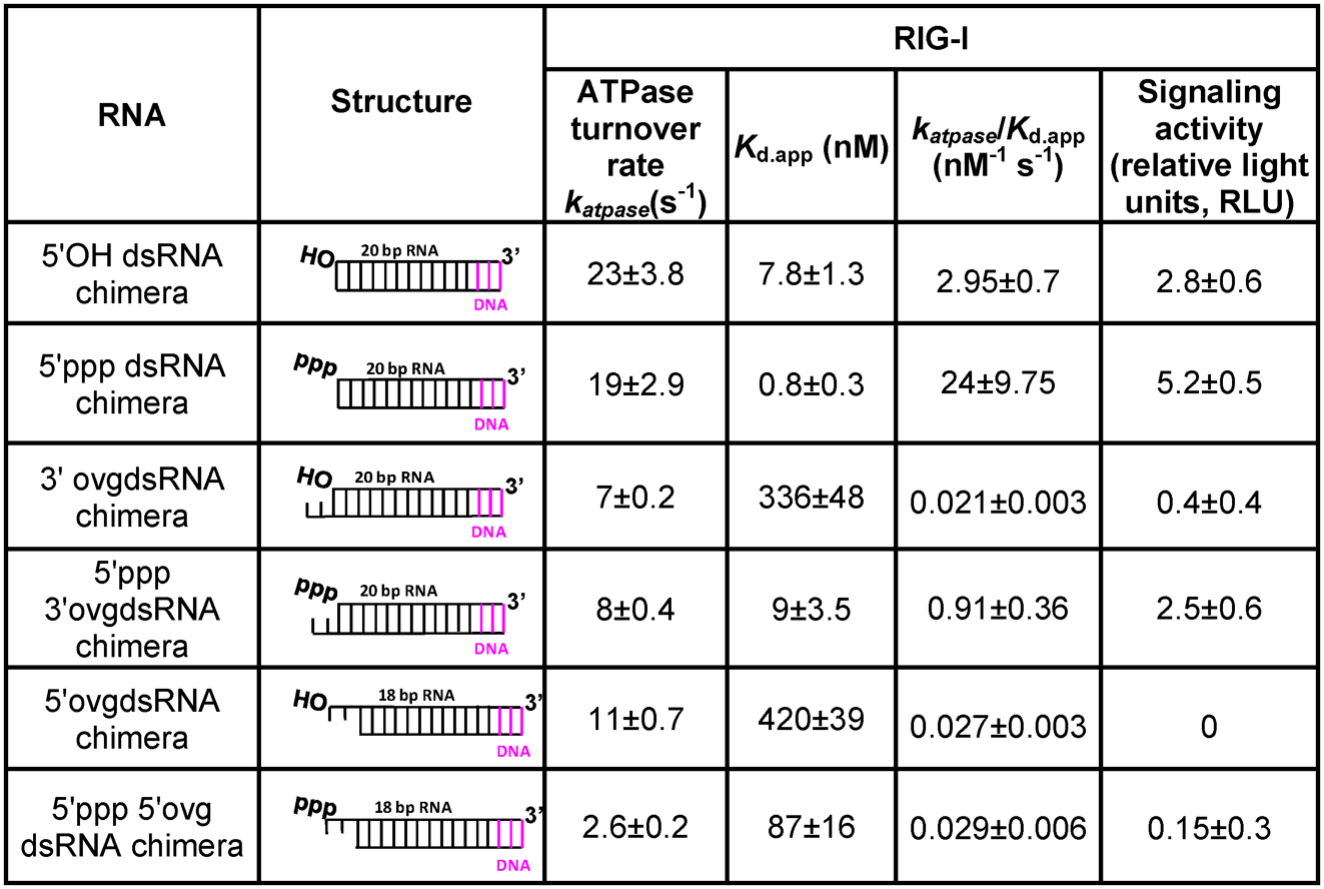					

**Figure 6.**
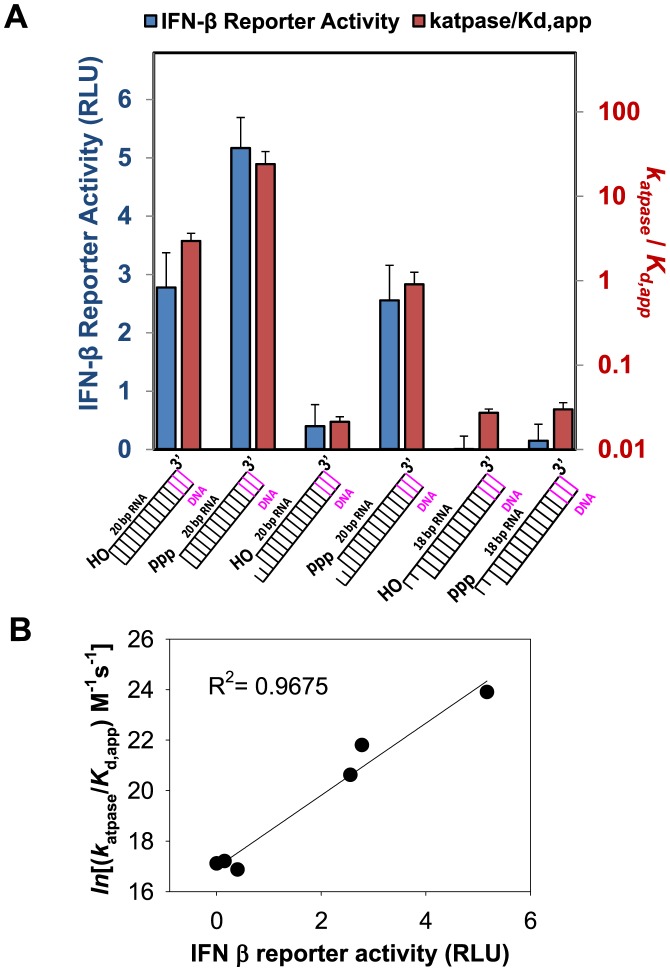
Signaling Activity of dsRNA with various end-modifications. **(A)** The *k*_atpase_/RNA *K*_d,app_ of each RNA ligand (blue bars) is plotted alongside their respective IFN-β promoter response elicited in signaling assays (red bars). Error bars are SEM of data collected from quadruplicate sets. **(B)** Correlation between signaling and logarithm of *k*_atpase_/*K*_d_ of RNAs.

The blunt-ended 5′ppp RNA showed the highest signaling activity, followed by the 5′OH RNA and 5′ppp 3′ovg RNA whose signaling activity is only ≈2-fold lower relative to the 5′ppp RNA (Figure 6A). The rest of the 23 bp non-blunt-ended dsRNAs have much lower signaling activity relative to the 5′ppp RNA; the 5′OH 3′ovg RNA has ≈10-fold lower signaling, the 5′ppp 5′-ovg RNA has ≈30-fold lower signaling and the 5′OH 5′ovg RNA has undetectable signaling activity. We also tested the CARD2-Hel2i interface mutant in cell-based signaling assays. Although constitutive activation of the RIG-I R109A has been reported (34), we did not observe this effect on our double mutant which showed significantly lower signaling relative to wt RIG-I (Supplementary Figure S6A). We think this is because R109 and neighboring residues are important for downstream events such as CARD oligomerization. For example, the double mutant (K108A/R109A) is defective in ubiquitin-mediated CARD oligomerization and signaling (35). Interestingly, another CARD2-Hel2i interface mutant where the mutation was made in the helicase domain (F539D) was constitutively active (24). This phenotype could be because of defects in RNA selectivity leading to activation by cellular RNAs.

To correlate the biochemical parameters of the RNAs with different end-modifications with their ability to stimulate RIG-I in cell-based signaling assays, we plotted the *k*_atpase_, *K*_d,app_ and *k*_atpase_/*K*_d,app_ of the 23-bp dsRNA against the signaling activity. We find no correlation between signaling and ATPase turnover rate (Supplementary Figure S6). This is interesting, because it has been suggested that the role of ATPase activity is to facilitate active dissociation of RIG-I from non-PAMP RNAs, thus preventing RIG-I from forming signaling competent complexes with such RNAs (32,36). This appears to be inconsistent with our observation showing that the ATPase activity of RIG-I weakens the binding affinity of only two RNAs, the 5′OH RNA and 5′ppp 3′ovg RNA, and both of these RNAs are signaling active. Moreover, the ATPase turnover rate of RIG-I complexes with non-blunt ended dsRNAs, most of which are signaling inactive, is lower (4–10-fold) than their blunt-ended counterparts.

We did find good correlation between signaling and ratio of ATPase turnover rate and *K*_d,app_ (logarithm of *k*_atpase_/*K*_d,app_) (Figure 6B) as well as a good correlation between signaling and RNA affinity (the logarithm of 1/*K*_d,app_) (Supplementary Figure S6). Under our cell-based assays, only RNAs between 1 and 10 nM *K*_d,app_ and 1–20 × 10^9^ M^−1^ s^−1^ *k*_atpase_/*K*_d,app_ show significant signaling activity (Table 3). Thus, RNA ligands that bind with a high affinity and stimulate high ATPase turnover rate are the best signaling substrates.

## DISCUSSION

Activation of RIG-I is an essential step in establishing an antiviral response in the cell. Equally important is assuring that RIG-I is activated selectively by non-self viral RNAs and not by self RNAs present in the cytoplasm. Insights into the molecular mechanism for how RIG-I selects non-self dsRNA ends from self were obtained by studying the binding affinity and ATPase activity of dsRNA with various end-modifications using the full-length RIG-I and different domain constructs of RIG-I. Our studies show that all the domains of RIG-I play a role in RNA selection. The C-terminal RD has high affinity for blunt-ended dsRNAs with and without 5′ppp, compared to non-blunt ended RNAs, and aids in RNA selection. The helicase domain provides additional binding sites for dsRNA and is critical for high affinity binding of 5′ppp dsRNAs carrying 3′-overhang. Most interestingly, CARDs regulate RNA affinity and ATPase turnover activity; even though, there is no evidence that CARDs interact with RNA.

The regulation by CARDs involves an allosteric mechanism whereby interactions between CARDs and Hel2i subdomain stabilizes the autoinhibited state of RIG-I, which is not efficient in RNA binding and ATPase activation. Deletion of CARDs increases both the RNA binding affinity and the ATPase activity, such that all types of RNAs including non-blunt-ended dsRNAs bind tightly and stimulate the ATPase rates of the Helicase-RD. This indicates that in the autoinhibited state, CARDs are selectively preventing binding of ssRNAs, dsRNA stem structures and dsRNA with 5′-overhang, but not hindering binding of blunt-ended dsRNA and dsRNA with 3′-overhang. The data also imply that once CARDs are released from its interactions with the helicase domain, RIG-I binds with a high affinity to all types of RNAs. Deleting CARDs drastically reduces RNA selectivity for blunt-ended 5′ppp dsRNA over non-blunt ended dsRNAs from ≈3000-fold to ≈150-fold. Similarly, RNA selectivity is reduced from ≈3000-fold to ≈750-fold when the CARD2-Hel2i interface is mutated. Thus, the CARD2-Hel2i interface known for its role in autoinhibiting RIG-I, is also involved in self *versus* non-self RNA selection.

We find it interesting that the 5′ppp 3′ovg RNA binds tightly to RIG-I and activates signaling. To understand the structural basis for tight binding of the 5′ppp 3′ovg RNA, we modeled the 3′overhang onto the blunt-ended dsRNA helicase-RD complex (3TMI). The helicase-RD contains a pore with basic amino acids at the interface between the RD and Hel1 domains, where the 3′ovg was accommodated with only minor additional protein rearrangements (Supplementary Figure S7). This explains why siRNAs containing 2-nt 3′-ovg and 5′ppp are able to activate RIG-I (37) and why they are such great tools for both silencing genes and activating the immune response, potentially treating certain cancers (38). We also find that accommodating the 5′ovg in dsRNA will require a larger conformational change in RIG-I, because of the intimate interactions of the RD domain with the 5′-blunt end. This explains why certain viruses such as Arena virus introduce 5′-ovg during replication to evade RIG-I mediated immune response. These structures are thought to act as RIG-I decoys and trap RIG-I in an inactive complex (39,40). We show that RIG-I also binds to dsRNA stem mimics albeit weakly with ≈600 nM *K*_d_, but this could be tight enough to form an initial complex to aid in the search for the correct RNA end by translocation (41). This flexibility in binding to RNA ligands apart from its characterized PAMP ligand is not unique to RIG-I. IFIT family of proteins play a key role in recognition of 5′ppp ssRNAs and seem to have a complementary role to RIG-I (42,43). Similar to the evidence provided here, a recent study showed that IFIT5 can bind to RNA ligands with 5′-end modifications other than 5′ppp and this flexibility in recognizing multiple RNA ligands increases the functionality of these immune receptors (44).

**Figure 7.**
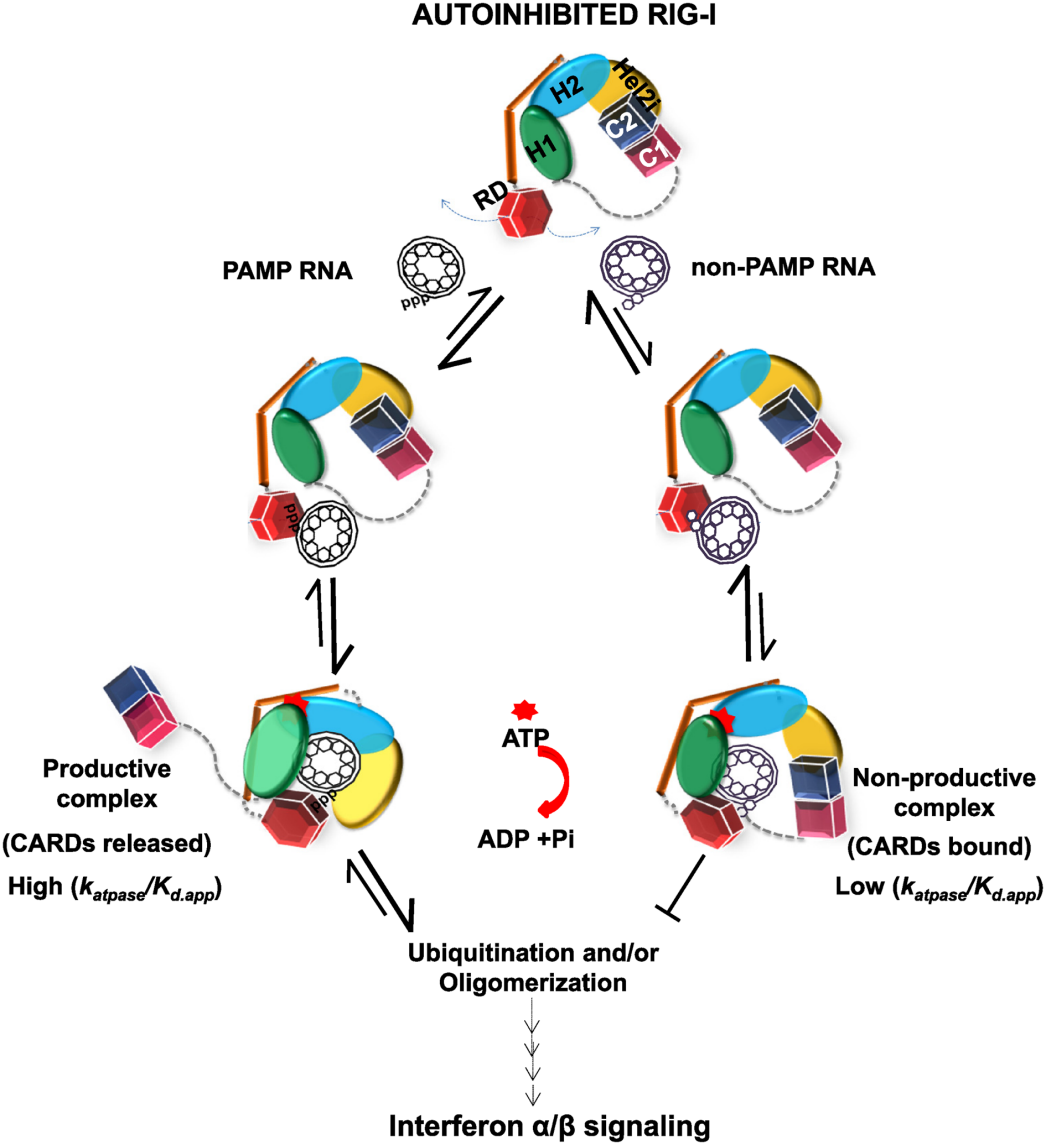
Model of RNA selectivity and RIG-I activation. RIG-I exists in the autoinhibited state in the absence of RNA binding where the CARD2 (C2, blue) is interacting with the Hel2i (yellow). PAMP and non-PAMP RNAs are sampled initially by RIG-I's C-terminal RD (red). PAMP RNAs (e.g. 5′ppp blunt-ended dsRNAs) bind with a high affinity (pathway on the left) to RD and the helicase domains (Hel1, Hel2, Hel2i), and the complex undergoes an induced-fit to disrupt the CARD2-Hel2i interactions by rotation of the Hel2-Hel2i subdomains, which results in new interactions with the dsRNA. These complexes have high affinity and high ATPase activity, with the potential to and undergo downstream events like ubiquitination and/or oligomerization, which ultimately leads to signaling. The RD and helicase binds weakly to non-PAMP RNAs (e.g. dsRNAs with 5′-overhang) (pathway to the right), which results in non-productive complexes with low affinity and low ATPase activity that are unable to signal, and eventually dissociate.

Based on crystal structures (19,24,45,46) and our biochemical studies of non-blunt ended dsRNAs, we propose the following model of self *versus* non-self RNA selection by RIG-I (Figure 7). We propose that, in the autoinhibited state, RIG-I samples RNAs in the cytoplasm via its RD and helicase subdomains. The RD may sample RNA stems in folded RNA structures or short single-stranded RNA, but these structures bind poorly and dissociate before the helicase domain can fully engage the RNA. This provides the first layer of RNA selectivity. Blunt-ended dsRNAs with and without 5′ppp and 5′ppp 3′-overhang dsRNAs bind tightly and the intrinsic binding energy appears to be enough to break CARD2-Hel2i interface. Movement of the CARDs allows rotation of the Hel2-Hel2i and new contacts with dsRNA and ATP to generate an ATPase-activated intermediate that can progress to the next step. In case of non-blunt ended dsRNAs, the energetic cost of breaking the CARD2-Hel2i interface is greater than that gained from RNA binding and these RNAs either populate low amounts of ATPase productive complexes or form non-productive complexes with low ATPase activity. Thus, an induced-fit conformational change provides a second layer of RNA selection. The steps after ATP and RNA binding, e.g. CARDs ubiquitination, oligomerization etc. add more layers of RNA selection to filter out self RNAs (35,41,47,48). Having multiple steps between RNA binding and signal transmission increases the selectivity beyond differences observed in intrinsic RNA binding affinities. This model invoking non-productive complexes with non-PAMP RNAs that can bind and activate ATPase with less efficiency, refines a previously suggested model of RNA selection by RIG-I (49).

Our studies show that molecular switches that are normally involved in autoinhibition are also involved in RNA selection. Autoinhibition mechanisms involving interdomain reorganization is not unique to RIG-I, but generally found in cellular molecular switches. One such example that closely relates to RIG-I's mechanism is the APAF-1 protein (Apoptotic protease activating factor 1), which initiates the intrinsic apoptotic signaling pathway (50,51). The C-terminal WD40 repeat domain in APAF-1 serves the dual function of maintaining autoinhibition and sensing cytochrome c. In APAF-1, a second layer of autoinhibition is provided by CARD-helicase domain interface (analogous to the CARD2-Hel2i interface in RIG-I), and this autoinhibition barrier is overcome by exchange of ADP with ATP. We propose that regulatory mechanisms, where certain structural elements, such as the CARD2-Hel2i interface in RIG-I, serve dual roles of regulating receptor activation as well as aid in ligand selection, are general. Many innate immune receptors including Toll-like receptors and NOD like receptors are regulated by such autoinhibition mechanisms (52), which may also be used for discriminating self *versus* non-self molecules.

## SUPPLEMENTARY DATA

Supplementary Data are available at NAR Online.

SUPPLEMENTARY DATA
