# Supplementary material for: The autoinhibitory CARD2-Hel2i Interface of RIG-I governs RNA selection
Source: Nucleic Acids Res. 2015 Nov 26;44(2):896–909. doi: 10.1093/nar/gkv1299 (PMC4737149; doi:10.1093/nar/gkv1299)
Supplement: SUPPLEMENTARY DATA [file supp_44_2_896__index.html]

The autoinhibitory CARD2-Hel2i Interface of RIG-I governs RNA selection — The autoinhibitory CARD2-Hel2i Interface of RIG-I governs RNA selection — SUPPLEMENTARY DATA 

# The autoinhibitory CARD2-Hel2i Interface of RIG-I governs RNA selection

## SUPPLEMENTARY DATA

- SUPPLEMENTARY DATA
