# Supplementary material for: The autoinhibitory CARD2-Hel2i Interface of RIG-I governs RNA selection
Source: Nucleic Acids Res. 2015 Nov 26;44(2):896–909. doi: 10.1093/nar/gkv1299 (PMC4737149; doi:10.1093/nar/gkv1299)
Supplement: SUPPLEMENTARY DATA [file supp_gkv1299_nar-02361-r-2015-File012.pdf]

SUPPLEMENTARY DATA

The autoinhibitory CARD2-Hel2i Interface of RIG-I governs RNA selection.

Anand Ramanathan[1,†], Swapnil C Devarkar[1,†], Fuguo Jiang[2], Matthew T. Miller[2], Abdul G. Khan[2], Joseph Marcotrigiano[2*] , Smita S Patel[1*].

[1] Robert Wood Johnson Medical School, Department of Biochemistry and Molecular Biology, Rutgers University, Piscataway, New Jersey 08854, USA.

[2] Center for Advanced Biotechnology and Medicine, Department of Chemistry and Chemical Biology, Rutgers University, Piscataway, New Jersey 08854, USA.

[†] These authors contributed equally to the paper as first authors.

[*] To whom correspondence should be addressed.

S.S.P (Email: patelss@rutgers.edu); J.M. (Email: jmarco@cabm-new.rutgers.edu)

SUPPLEMENTARY FIGURES

Figure S1

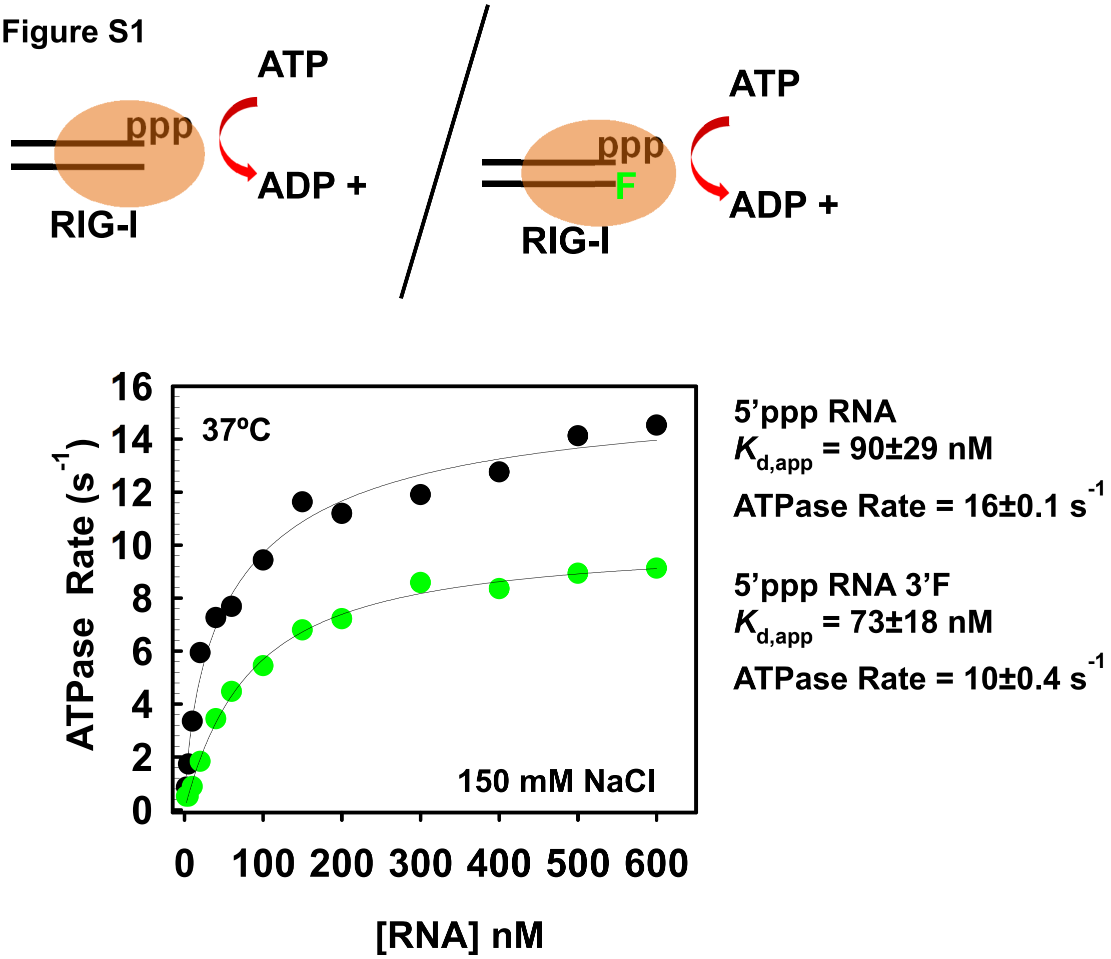

Figure S1, Related to Figures 2-6, Effect of fluorescein probe on RNA binding by RIG-I. ATPase based titration experiment; RIG-I (5 nM) was titrated with 5'ppp RNA with or without the 3' fluorescein probe. A time course ATPase assay was performed at different RNA concentrations at 37°C in Buffer A containing 150mM NaCl. A fit to quadratic equation revealed $K_{d,app}$ of 90±29 nM for 5'ppp RNA without probe (black circles) and 73±18 nM for 5'ppp RNA with 3' fluorescein probe. Error is standard error from fit.

Figure S2

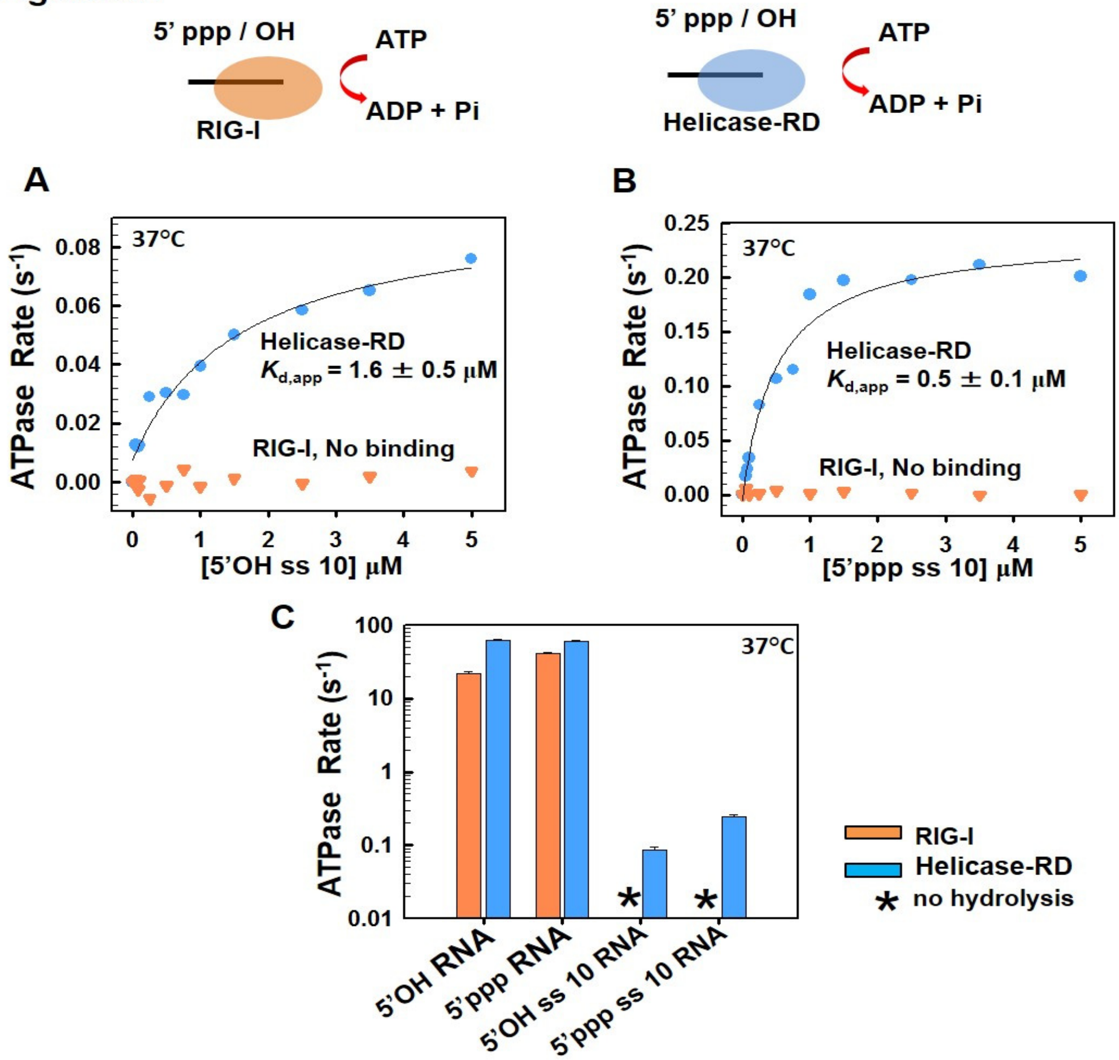

Figure S2. Related to Figure 2 and 4;RIG-I and Helicase-RD interaction with ssRNA. ATPase based titration experiment; RIG-I (100nM) (orange inverted triangles) and Helicase-RD (100nM) (blue circles) was titrated with 5’OH ss10 RNA **(A)** and 5’ppp ss 10 RNA **(B).**RIG-I shows little or no affinity for 5’OH ss10 RNA or 5’ppp ss 10 RNA. Helicase-RD binds 5’OH ss10 RNA $K_{d,app}$ of 1.6±0.5 μM and 5’ppp ss 10 RNA with $K_{d,app}$ of 0.5±0.1 μM. A time course ATPase assay was performed at different RNA concentrations at 37°C in Buffer A. Data was fit to hyperbolic equation and standard error from fit is shown. **(C)** Comparison of the maximum ATPase Rate s^{-1} of RIG-I (orange bars) and Helicase-RD (blue bars) in presence of 5’OH ss10 RNA, 5’ppp ss10 RNA, 5’OH RNA and 5’ppp RNA. A time course ATPase assay was performed using 5nM protein (for dsRNA experiment) or 100nM protein (for ss RNA experiment), 1mM ATP and 1 μM RNA at 37°C in Buffer A. Data was fit to hyperbolic equation to obtain the ATPase Rates and standard error from fit is shown. No hydrolysis (asterisk).

Figure S3

A

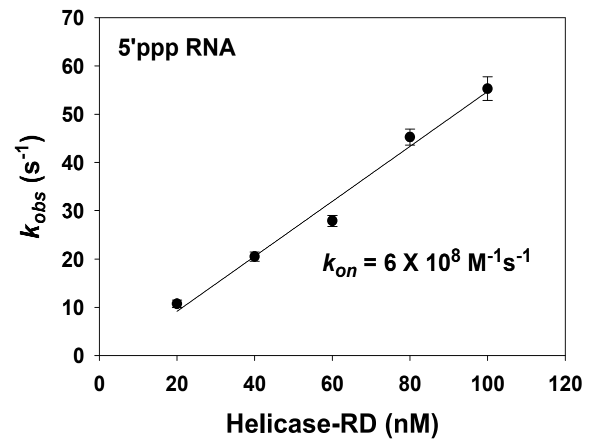

B

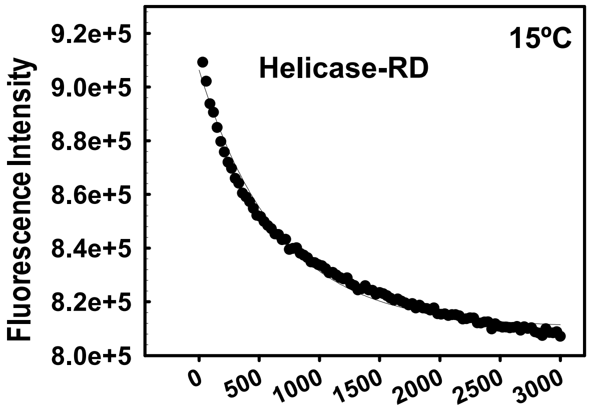

Figure S3, Related to Figure 4, Kinetics of 5'ppp RNA interaction with helicase-RD. (A) Fluorescently labeled 5'ppp RNA (10 nM) was mixed with increasing concentrations of helicase-RD (20-100 nM). The observed rate (k_{obs}) was plotted as a function of protein concentration to obtain the k_{on}, **(B)** Equimolar concentrations (50 nM) of the protein and RNA were preincubated and then mixed with 2mM ATP. A 6 fold excess of unlabeled RNA was used as "trap". Fluorescence intensity was plotted as a function of time to obtain the k_{off}. Detailed experimental set-up in Supplemental Experimental Procedures.

Figure S4

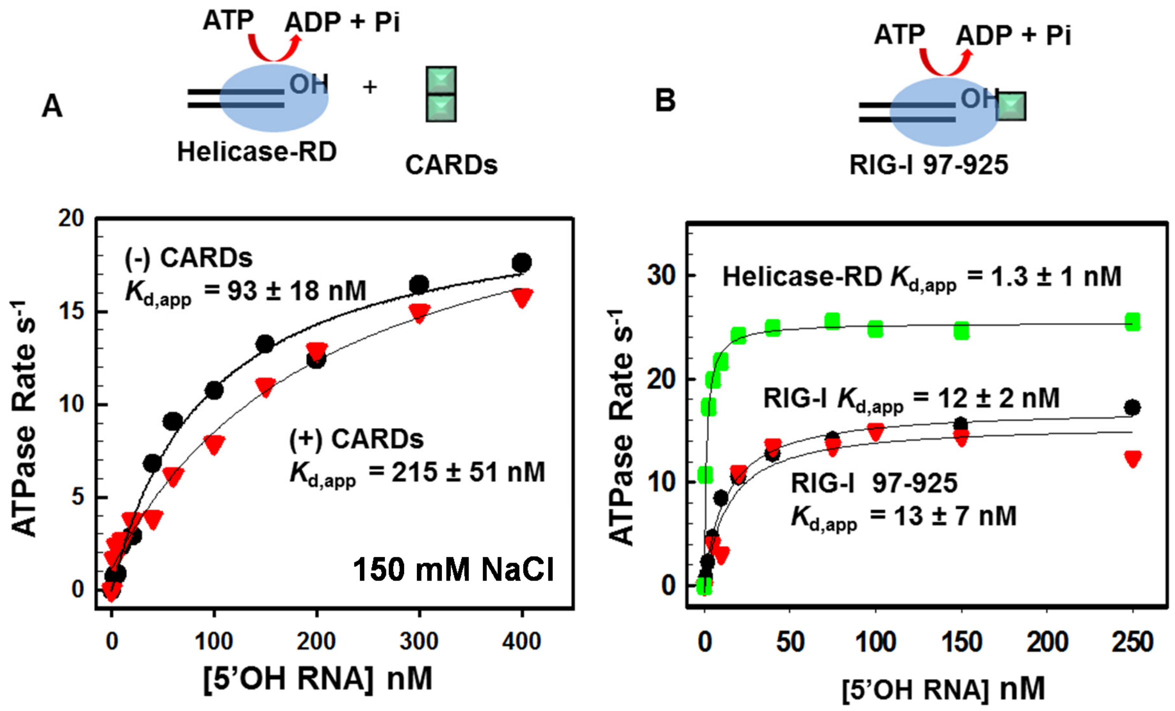

Figure S4: Related to figure 4, RNA binding is regulated by CARDs and specifically by CARD2. (A) Helicase-RD (5 nM) was titrated with increasing concentrations of 5'OH RNA without CARDs (black circles) or with 3 µM RIG-I CARDs added in trans (red inverted triangles) and ATPase rates were measured at 37°C in Buffer A containing 150 mM NaCl. Data were fit to hyperbolic equation to obtain the $K_{d,app}$. **(B)** RIG-I, Helicase-RD or RIG-I 97-925 (containing 2nd CARD) at 5 nM protein was titrated with increasing concentrations of 5'OH 18 bp RNA and ATPase rates were measured at 37°C in Buffer A. The $K_{d,app}$ was obtained by fitting to the hyperbolic equation.

Figure S5

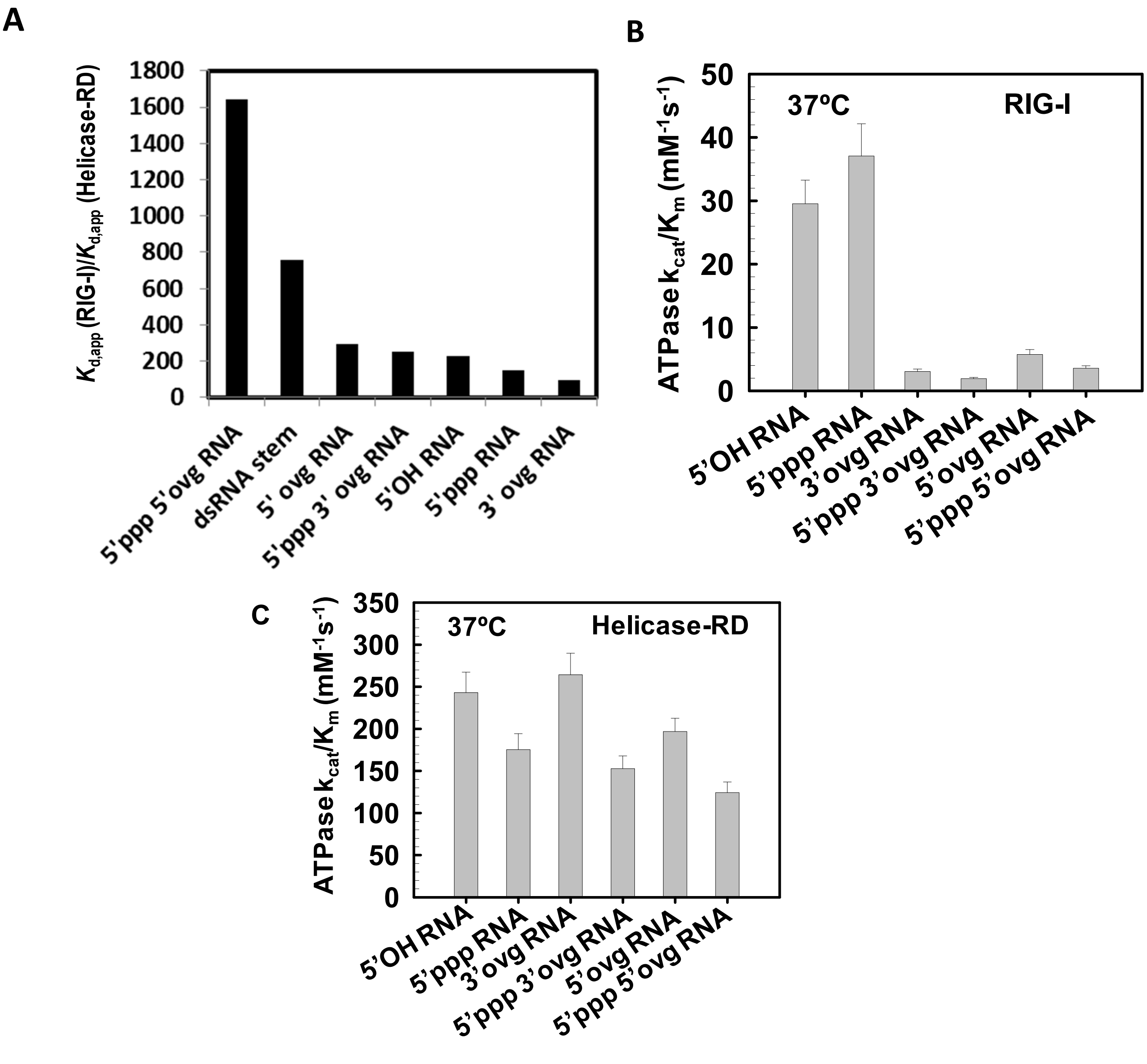

Figure S5: Related to figure 2 and 4, RNA $K_{d,app}$ ratio, ATPase k_{cat}/K_m values of RIG-I (wt) and Helicase-RD for the various RNA ligands. (A) The ratio of the $K_{d,app}$ values of RIG-I and Helicase-RD obtained by dividing $K_{d,app}$ RIG-I/ $K_{d,app}$ Helicase-RD. **(B-C)** k_{cat}/K_m values for RNAs carrying various end modifications were measured at 37^0C for RIG-I (wt) and Helicase-RD respectively.

Figure S6

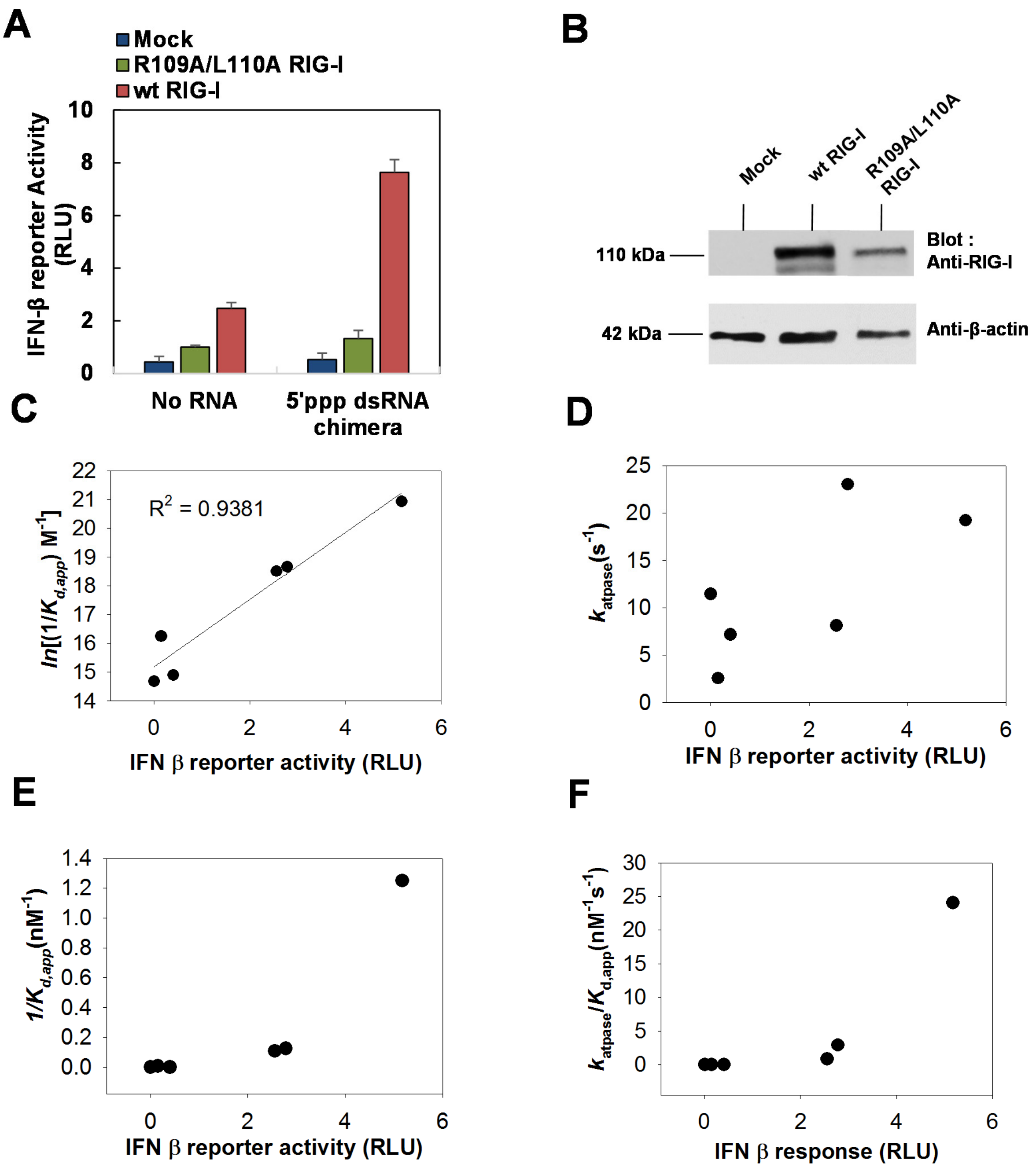

Figure S6: **Related to Figure 6**, (A) IFN-β reporter activity of mock transfected (blue bars), R109A/L110A (green bars) and (wt) RIG-I transfected (red bars) HEK293T cells for 5'ppp dsRNA. (B) Western Blots showing expression of R109A/L110A and wt RIG-I in transfected HEK293T cells(C-F) Correlation between signaling and various biochemical parameters.

Figure S7

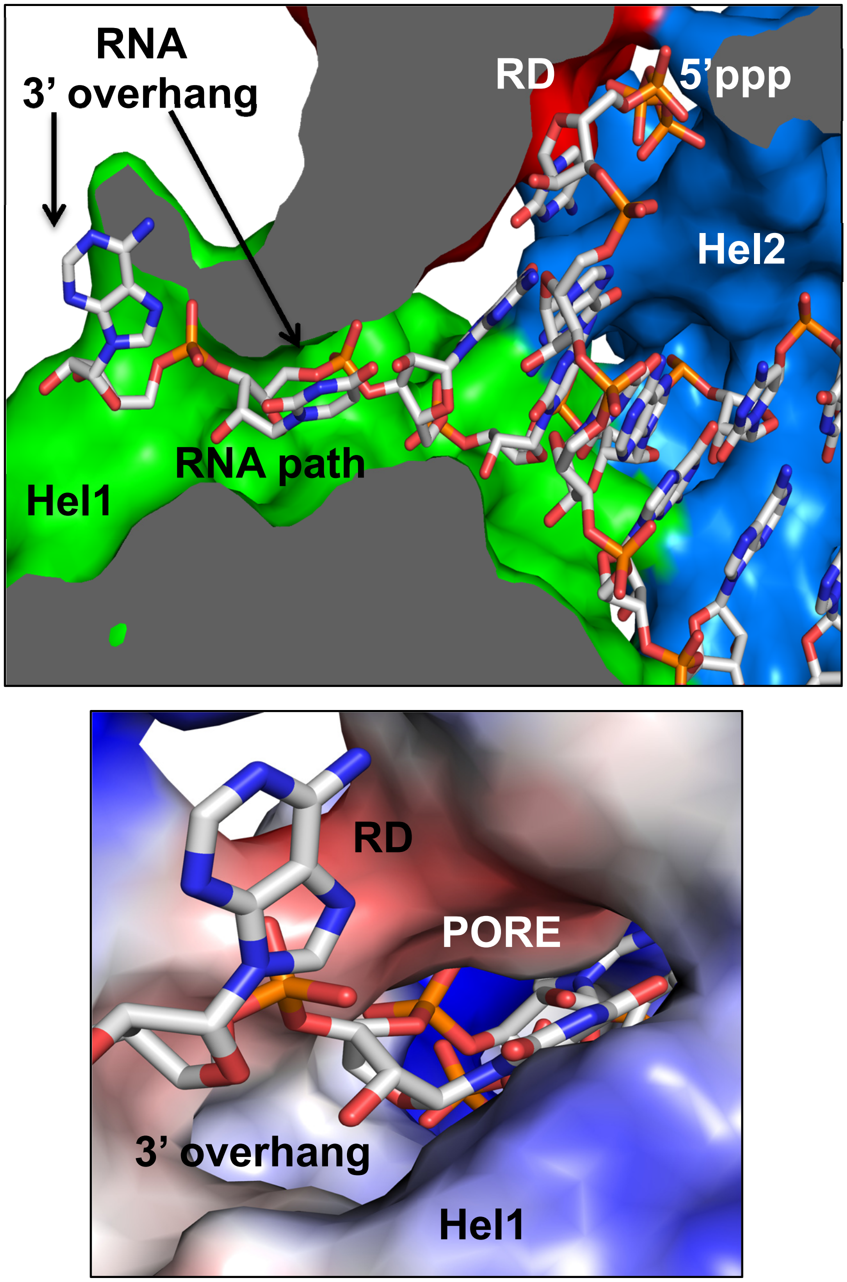

Figure S7: Related to Figure 2, Model of RIG-I with 5'ppp 3'ovg RNA. (A) The RNA path of the 3' overhang near the 3' end of the double stranded RNA and 5'ppp of the complementary strand interacting with RD. **(B)** Overhanging 3' nucleotides in the basic patch of amino acids in the pore created between Hel1 and RD. Models were constructed using the Helicase-RD/dsRNA bound structure (3TMI). The dsRNA was replaced with 5'ppp 3'ovg RNA used in this study.

SUPPLEMENTAL TABLES.

Table S1. Sequences of RNA

Sequence	RNA name	RNA end	Source
5’pppCGUGAGACAU 3’	5’ppp 10 ss	5’ppp	Biosynthesis
5’CGUGAGACAU 3’	5’OH 10 ss	5’OH	Dharmacon
5’AUGUCUCACG 3’	5’ OH 10 ss comp	5’OH	Dharmacon
5’AUGUCUCACG (F) 3’	3’ F10 ss	5’OH, 3’F	Dharmacon
5’ GUCUCACGUA (F) 3’	3’ovg 3’ F10 ss	5’OH, 3’F	Dharmacon
5’(F) AUGUCUCACG 3’	5’ F10 ss	5’F	Dharmacon
5’(F) UCAUGUCUCA 3’	5’ ovg 5’ F10 ss	5’F	Dharmacon
5’GAAUAUAAUAGUGAUAUUAUAUUC 3’	5’OH HP	5’OH	Dharmacon
5’pppGAAUAUAAUAGUGAUAUUAUAUUC 3’	5’ppp HP	5’ppp	Biosynthesis
5’UUUUUGAAUAUAAUAGUGAUAUUAUAUUC UUUUU 3’	dsRNA Stem	5’OH	Dharmacon
5’GGCUGUAACUGUGGUCUA 3’	5’ 18 ss	5’OH	Dharmacon
5’ UAGACCACAGUUACAGCC 3’	3’ 18 ss	5’OH	Dharmacon
5’GGAGAGUACCUCCAACGCCAdGdCdG 3'	5'OH RNA 3’Chi 23ss	5’OH	Dharmacon
5’pppGGAGAGUACCUCCAACGCCAdGdCdG 3'	5'ppp RNA 3’Chi 23ss	5'ppp	Biosynthesis
5'dCdGdCUGGCGUUGGAGGUACUCUCC 3'	5'OH RNA 5’Chi 23ss	5’OH(DNA)	Dharmacon
5'dCdGdCUGGCGUUGGAGGUACUCUCCUA 3'	5'OH RNA 5’Chi 25ss	5’OH(DNA)	Dharmacon
5'dCdGdCUGGCGUUGGAGGUACUCU 3'	5'OH RNA 5’Chi 21ss	5’OH(DNA)	Dharmacon

ppp - triphosphate, OH - hydroxyl, ds - double stranded, ss - single stranded, HP-Hairpin, F-Fluorescein, ovg - 2 nucleotide overhang, comp – complementary, dN – deoxyribonucleotide, Chi - chimera.

Table S2. ATPase Rates and $K_{d,app}$ of RIG-I for the Stem and Hairpin RNAs.

RNA	Structure	RIG-I		Helicase-RD	
		ATPase Rate (s^{-1}) 37ºC	$K_{d,app}$ (nM) 37ºC	ATPase Rate (s^{-1}) 37ºC	$K_{d,app}$ (nM) 37ºC
dsRNA stem	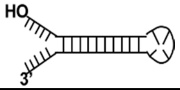	4±0.3	629±165	47±1	0.5±0.4
5'OH HP	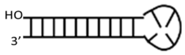	50±1	39±4	67±2	2±0.4
5'ppp HP	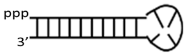	37±1	6±0.7	51±2	3±0.4

Table S3. **ATPase K_m, k_{cat}, and k_{cat}/K_m of RIG-I and Helicase-RD for the different RNAs**

RNA	RIG-I			Helicase-RD		
	k_{cat}/K_m ($mM^{-1}s^{-1}$) 37ºC	ATP K_m (mM)	ATPase k_{cat} (s^{-1})	k_{cat}/K_m ($mM^{-1}s^{-1}$) 37ºC	ATP K_m (mM)	ATPase k_{cat} (s^{-1})
5'OH RNA	30±4	0.74±0.08	22±1	243±24	0.26±0.025	63±2
5'ppp RNA	37±5	1.3±0.16	49±3	175±19	0.34±0.034	60±2
3' ovg RNA	3±0.4	0.78±0.08	2.3±0.1	264±26	0.16±0.014	41±1
5'ppp 3' ovg RNA	2±0.2	1.0±0.1	1.9±0.09	153±15	0.21±0.02	32±1
5' ovg RNA	6±0.8	0.51±0.06	2.9±0.1	197±16	0.14±0.04	27±0.5
5'ppp 5'ovg RNA	4±0.4	0.54±0.05	4±0.1	124±13	0.2±0.02	25±0.7

(The above data was collected at 37ºC)

Table S4. **RNA selectivity of RIG-I, Helicase-RD and RIG-I R109AL110A for 5'ppp RNA over other RNAs**

RNA	RIG-I selectivity	Helicase-RD selectivity	R109AL110A Selectivity
5'OH RNA	0.0140	0.0314	0.5
5'ppp RNA	1	1	1
5'OH 3' ovg RNA	0.0003	0.0013	0.0007
5'ppp 3' ovg RNA	0.0005	0.0092	0.0028
5'OH 5' ovg RNA	0.0002	0.0023	0.0008
5'ppp 5'ovg RNA	0.0005	0.015	0.0012
dsRNA stem	0.0002	0.0025	0.0011

$$\mathbf{RNA\ Selectivity} = \frac{\mathbf{k_{atpase}/K_{d,app}\ of\ an\ RNA\ ligand}}{\mathbf{k_{atpase}/K_{d,app}\ of\ an\ 5'\ \ ppp\ dsRNA}}$$

SUPPLEMENTAL EXPERIMENTAL PROCEDURES

Stopped-Flow Measurements of '*on'* rates

Real time measurements of the *on* and *off* rates were carried out using a T-scheme KinTek stopped-flow setup (Model 2003)(1). 5'ppp RNA was labeled at the 3' end (strand not carrying the 5'ppp moiety) with a fluorescein probe and used for these assays. The fluorescence intensity of the fluorescein probe was detected in real-time with the use of long-pass 515 nm filter. For the on-rate measurements, a fixed concentration of fluorescently labeled RNA was mixed with increasing excess concentrations of helicase-RD pre-incubated with 1.5 mM ATP. The time-course of the fluorescence intensity was fitted to the following exponential equation, to get an observed rate (k_{obs}).

$$Y = Ae^{-k_{obs}t} + C \qquad \text{Equation.S1}$$

where, A is the amplitude and k_{obs} is the observed rate for the protein concentration being used. The observed rates were then plotted against the corresponding concentrations of the protein. The plot was fit to a linear equation to get the slope of the line which corresponds the the 'k_{on}' or the *on* rate.

Off rate Measurement

Off rates of the Helicase-RD-5'ppp RNA 3'F complex were determined using the Fluoromax-4 spectrofluorometer (Horiba). Equimolar concentrations (50 nM) of the protein and RNA were preincubated and then mixed with 2mM ATP. The fluorescence intensity of the fluorophore was measured over a period of 60 min by excitation at 495 nm and detecting emission at 515 nm. The cuvette was maintained at 15°C and readings were taken every 60 seconds. A six-fold excess of non-fluorescent RNA was added after the initial reading as a 'trap' and the change in fluorescence intensity was plotted as a function of time and fitted to the following exponential equation to get the *off* rate.

$$y = y0 + Ae^{-kt} \qquad \text{Equation.S2}$$

where, A is the amplitude and k is the observed *off* rate.

Western Blot Analysis.

The protein expression levels of wt RIG-I and R109A/L110A RIG-I in HEK293T cells was assessed by Western blots. Transfected cells were lysed and an appropriate dilution of the lysates was run on a 4-15% gradient polyacrylamide gels. The samples were transferred to a nitrocellulose membrane (Hybond ECL, GE) and then probed with antibodies against RIG-I and β-actin (loading control). 5% milk solution was used for blocking and then probed with anti-RIG-I antibody (Adipogen) (1:1000 dilution in 1X PBST) or anti-β-actin antibody (Cell Signaling) (1:1000 dilution in 1X PBST) overnight. A secondary antibody against mouse IgGs conjugated with HRP (Cell Signaling) (1:15,000 dilution in 1X PBST) was used for detection of the primary antibodies. The bands were visualized using a chemiluminescent substrate kit (SuperSignal West Pico, Thermo Scientific) and appropriate exposure on an X-ray film (GeneMate).

Model of RIG-I Helicase-RD with 5'ppp 3'ovg RNA

The structure of Helicase-RD RIG-I bound to dsRNA, complexed with BeF_3 (3TMI) was used as the basis for the construction of a model in which the double stranded RNA was replaced with the 5'ppp 3' ovg RNA (refer Table S1 for sequence)used in this study into the RNA binding pocket. The modeling and geometry refinement were performed using Coot(2) and the figures and electrostatics were generated using Pymol(3).

References.

1. Bandwar, R.P. and Patel, S.S. (2002) The energetics of consensus promoter opening by T7 RNA polymerase. *Journal of molecular biology*, **324**, 63-72.
2. Emsley, P. and Cowtan, K. (2004) Coot: model-building tools for molecular graphics. *Acta Crystallogr D Biol Crystallogr*, **60**, 2126-2132.
3. DeLano, W.L. (2002). DeLano Scientific, San Carlos, CA, USA.
